# Correlation between anti-thyroid peroxidase antibody levels and diffuse thyroid uptake of ^18^F-fluorodeoxyglucose in Hashimoto’s thyroiditis: a retrospective study

**DOI:** 10.1186/s13044-018-0058-5

**Published:** 2018-10-16

**Authors:** Naoki Edo, Koji Morita, Miki Sakamoto, Tatsuro Kaminaga, Hiromi Edo, Erina Okamura, Masumi Ogawa, Kazuhisa Tsukamoto, Hiroko Okinaga, Toshio Ishikawa

**Affiliations:** 1 Department of Internal Medicine, Teikyo University School of Medicine, 2-11-1, Kaga, Itabashi, Tokyo, 173-0003 Japan; 2 Department of Radiology, Teikyo University School of Medicine, 2-11-1, Kaga, Itabashi, Tokyo, 173-0003 Japan; 3 Department of Radiology, Toho University Ohashi Medical Center, 2-22-36, Ohashi, Meguro, Tokyo, Japan

**Keywords:** Anti-thyroid antibody, Diffuse uptake, FDG-PET, Hashimoto’s thyroiditis

## Abstract

**Background:**

On ^18^F-fluorodeoxyglucose (FDG)-positron emission tomography (PET), diffuse uptake in the thyroid gland is often observed in patients with Hashimoto’s thyroiditis. In this study, we evaluated the factors associated with diffuse uptake by comparing Hashimoto’s thyroiditis patients with or without diffuse uptake in the thyroid.

**Methods:**

A retrospective study was conducted of 18 patients with Hashimoto’s thyroiditis who underwent blood tests, thyroid ultrasonography, and FDG-PET during the period from 2014 to 2015. The patients were divided into two groups: one with diffuse thyroid uptake (group 1, *n* = 13) and one without diffuse thyroid uptake (group 2, *n* = 5). Clinical and laboratory parameters, including maximum standardized uptake in the thyroid (SUVmax), which was defined as the higher value obtained in either the right or left thyroid lobe, were compared in the two groups.

**Results:**

The frequency of abnormal findings, such as a rough or heterogeneous pattern, was significantly higher in group 1 (*p* <  0.01), as were anti-thyroid peroxidase (TPO) antibody titers, anti-thyroglobulin (Tg) antibody titers, and SUVmax (*p* <  0.01). The frequency of hypothyroidism did not differ significantly in the two groups. Anti-TPO and anti-Tg titers were positively correlated with SUVmax (*r* = 0.856, *p* <  0.01 and *r* = 0.821, *p* <  0.01, respectively); in univariate analysis, anti-TPO titer was predictive of SUVmax (*p* <  0.01).

**Conclusions:**

The results of the current study suggest that Hashimoto’s thyroiditis patients with high titers of anti-thyroid antibodies are likely to exhibit intense diffuse FDG uptake in the thyroid, and that thyroid function may be clearly impaired, even in the presence of mild FDG uptake in the thyroid.

## Background

Positron emission tomography (PET) with ^18^F-fluorodeoxyglucose (FDG) is a functional imaging technique known as FDG-PET that exploits the typically increased rate of glycolysis in specific cell types, such as malignant tumors and inflammatory tissue [1, 2]. Its use as a method of detecting malignant tumors and foci of infection has recently become more frequent.

With regard to the thyroid, a normal thyroid gland is not visible or shows only low-grade FDG uptake on FDG-PET [3, 4]. However, the increasing use of FDG-PET has led to incidental identification of patients with increased FDG uptake in the thyroid gland. FDG uptake in the thyroid predominantly conforms to one of two patterns: focal and diffuse. The prevalences of incidental focal and diffuse uptake in the thyroid reportedly vary from 0.1 to 4.8% and 0.1 to 4.5%, respectively [5]. Focal uptake in the thyroid suggests the possibility of a malignant tumor, such as papillary thyroid carcinoma, although most well-differentiated thyroid carcinomas are relatively slow-growing and can be FDG-negative [6, 7]. In contrast, diffuse FDG uptake is typically considered to be a result of benign conditions, such as Hashimoto’s thyroiditis or Graves’ disease, among others [8, 9]; however, there have been few reports of diffuse uptake. Furthermore, among patients with Hashimoto’s thyroiditis, not all patients exhibit diffuse uptake.

In the current study, we evaluated the factors associated with diffuse uptake by comparing Hashimoto’s thyroiditis patients with or without diffuse uptake in the thyroid.

## Methods

### Patients

Records of 18 consecutive attendees (five men, 13 women) with Hashimoto’s thyroiditis who underwent blood tests, thyroid ultrasonography (US), and FDG-PET at our hospital during the period from 2014 to 2015 were examined retrospectively. Clinical diagnosis of Hashimoto’s thyroiditis was made based on the elevation of either anti-thyroid peroxidase (TPO) antibody titer or anti-thyroglobulin (Tg) antibody titer. Additionally, the possibility of neoplastic diseases was considered highly unlikely based on clinical symptoms and ultrasonographic findings, although we did not perform histopathological examinations. Details of the subjects are presented in Table 1. The patients were divided into two groups, one with diffuse thyroid uptake (group 1, *n* = 13) and one without diffuse thyroid uptake (group 2, *n* = 5). Clinical and laboratory parameters were investigated, including the maximum standardized uptake value (SUVmax) in the thyroid. The study was approved by the institutional review board of Teikyo University Hospital, Japan (18–036), and the need for written informed consent was waived.

**Table 1:** Details of the subjects

Subject number	Age, sex	Findings in thyroid ultrasonography	TSH (μIU/mL)	FT4 (ng/dL)	FT3 (pg/mL)	L-T4 replacement dose (μg/day)	Tg-Ab (IU/mL)	TPO-Ab (IU/mL)	SUV max	Interval Between FDG-PET and thyroid test (days)
Diffuse uptake										
1	51 F	Hetero, Hypo	22.43	1.06	2.20	0	143	22	3.322	13
2	78 F	Hetero	6.73	0.78	2.08	0	> 4000	487	8.125	11
3	68 F	Hetero, Hypo	73.06	0.57	2.37	25	> 4000	16	3.741	28
4	59 F	Hetero	6.43	0.93	3.21	0	n.a.	184	6.070	64
5	80 M	Rough, Hetero	28.59	1.07	2.56	0	> 4000	> 600	5.714	3
6	86 F	Hetero	21.04	0.68	1.75	12.5	476	53	3.354	1
7	64 F	Hetero	3.79	1.32	1.16	0	122	29	2.923	1
8	66 M	Hetero, Hypo	8.45	0.93	2.64	0	> 4000	> 600	6.165	21
9	78 F	Rough, Hetero	87.87	0.50	1.50	25	n.a.	> 600	12.023	4
10	41 F	Rough, Homo	46.21	0.94	1.58	125	626	> 600	9.985	3
11	43 F	Hetero	3.24	1.31	2.49	0	537	332	4.113	7
12	67 F	Rough, Hetero	25.11	0.94	3.04	0	2940	184	3.340	24
13	63 M	Rough, Hetero	266.1	0.19	1.16	0	1360	72	3.868	19
Without diffuse uptake										
14	74 F	Homo	0.033	1.23	3.12	0	147	10	2.492	10
15	68 F	Homo	11.01	1.09	2.45	0	465	8	2.364	39
16	50 M	Homo	5.38	0.96	2.15	0	18	22	1.535	14
17	80 F	Homo	12.27	1.56	0.89	100	18	16	1.337	1
18	74 M	Fine, Homo	11.23	1.11	2.39	0	16	18	1.704	83

*F* female, *M* male, TSH serum thyroid stimulating hormone, *FT4* serum free T4, *FT3* serum free T3, *L-T4* levothyroxine, *Tg-Ab* anti-thyroglobulin antibody, *TPO-Ab* anti-thyroid peroxidase antibody, *SUVmax* maximum standardized uptake, *FDG* ^18^F-fluorodeoxyglucose, *PET* positron emission tomography, *F* female, *M* male, *Hetero* heterogenous, *Hypo* hypoechoic, *Homo* homogenous, *n.a.* not assessed

### Data analysis

Serum levels of thyroid stimulating hormone (TSH), free T4, Tg, anti-Tg antibody, and anti-TPO antibody were measured. Thyroid US was performed close to the time of FDG-PET examination. SUVmax in the thyroid was recorded as the higher value obtained from either the right or the left thyroid lobe.

### PET/computed tomography

All PET scans were performed on a PET/computed tomography (CT) system (Biograph 40 True Point, Siemens Health Care, Erlangen, Germany). FDG was supplied via a commercial delivery system (Nihon Medi-Physics Co., Ltd., Tokyo, Japan). All patients were fasted for at least 8 h before the injection of 165.5–352.5 MBq of FDG. Imaging was performed 1 h after injection and 2 h after injection. Three-dimensional scanning data were obtained from the top of the skull through to the pelvis, with a 3-min acquisition time per bed position. The studies were reconstructed using a vendor-supplied iterative reconstruction algorithm.

### Laboratory tests

Serum levels of TSH, free T3, and free T4 were measured by using a chemiluminescence enzyme immunoassay (Lumipulse Presto, Fujirebio Co., Tokyo, Japan). Serum Tg, anti-Tg titer, and anti-TPO titer were measured with an electrochemiluminescence immunoassay (Elecsys Tg, Elecsys Anti-TPO, and Elecsys Anti-Tg, respectively; Roche Diagnostics K. K., Tokyo, Japan). The reference ranges for these laboratory tests at our institution were 0.5–5.0 mIU/mL for TSH, 2.3–4.0 pg/mL for free T3, 0.9–1.7 ng/dL for free T4, 0–33.7 ng/mL for serum Tg, 0–27 IU/mL for anti-Tg titer, and 0–15 IU/mL for anti-TPO titer. US examinations were performed with an 8.0-MHz linear phased-array probe (Aplio-XG, Canon Medical Systems Corporation, Tokyo, Japan). FDG uptake was evaluated by two board-certified radiologists (TK and HE).

### Statistical analysis

All comparisons between the two groups were analyzed using the nonparametric Mann-Whitney U test. Differences in prevalence between men and women were evaluated by using the χ^2^ test. Correlations were assessed by using Spearman’s correlation coefficient analysis. All statistical analyses were performed with R version 3.4.1 (The R Foundation for Statistical Computing, Vienna, Austria). Two-tailed *p* values < 0.05 were considered significant.

## Results

### Comparison of clinical and laboratory parameters

Table 2 shows comparisons of clinical and laboratory parameters between patients with or without diffuse thyroid uptake of FDG. Anti-Tg titer and anti-TPO titer were significantly higher in group 1 than in group 2. Serum free T4 was also significantly higher in patients with diffuse thyroid uptake, but it was similar in groups 1 and 2 after excluding those undergoing replacement therapy consisting of levothyroxine. No significant differences in other parameters were detected. The patients in group 1 were more likely to exhibit diffusely swollen thyroids with a rough and/or heterogeneous pattern on thyroid US.

**Table 2:** Comparisons of clinical and laboratory parameters in the two groups

	Group 1: Patients with diffuse uptake	Group 2: Patients without diffuse uptake	*p*
*n* (female)	13 (10)	5 (3)	0.583
Serum TSH Median (minimum-maximum)	22.43 (3.24–266.1)	11.01 (0.033–12.27)	0.143
Serum TSH (only patients without replacement therapy) Median (minimum-maximum)	8.45 (3.24–266.1)	8.20 (0.03–11.23)	0.414
Serum free T3 Median (minimum-maximum)	2.20 (1.16–3.21)	2.39 (0.89–3.12)	0.921
Serum free T4 Median (minimum-maximum)	0.93 (0.19–1.32)	1.11 (0.96–1.56)	< 0.05
Serum free T4 (only patients without replacement therapy) Median (minimum-maximum)	0.94 (0.19–1.32)	1.10 (0.96–1.23)	0.247
Serum thyroglobulin Median (minimum-maximum)	1.465 (0.08–175.0)	3.435 (2.94–3.93)	0.533
Tg-Ab titer Median (minimum-maximum)	1360 (122–4000)	18 (16–465)	< 0.01
TPO-Ab titer Median (minimum-maximum)	184 (16–600)	16 (8–22)	< 0.01

*TSH* thyroid stimulating hormone, *Tg-Ab* anti-thyroglobulin antibody, *TPO-Ab* anti-thyroid peroxidase antibody

### Associations between thyroid SUVmax and thyroid autoantibodies

Associations between thyroid SUVmax and thyroid autoantibodies were evaluated in all subjects. Thyroid SUVmax was positively correlated with both anti-TPO titer (*r* = 0.856, *p* <  0.01) and anti-Tg titer (*r* = 0.821, *p* <  0.01). Notably, however, anti-TPO and anti-Tg titers were also strongly correlated with each other (*r* = 0.607, *p* <  0.05). Therefore, univariate analysis of SUVmax was performed for each autoantibody. Anti-TPO titer was a significant predictor of thyroid SUVmax in univariate analysis, but anti-Tg titer was not (Table 3). After transformation into Z-scores, anti-TPO titer yielded a β-coefficient of 0.851 (*p* <  0.01).

**Table 3:** Anti-TPO antibody titer and anti-Tg antibody titer univariate regression with SUVmax

1. Anti-TPO antibody				
Variate	*B* (S.E.)	β	t	*p*
Intercept	2.312 (0.546)		4.23	< 0.01
TPO-Ab titer	0.010 (0.002)	0.851	6.26	< 0.01
2. Anti-Tg antibody				
Variate	*B* (S.E.)	β	t	*p*
Intercept	2.949 (0.777)		3.80	< 0.01
Tg-Ab titer	0.001 (0.000)	0.390	2.10	0.06

*TPO-Ab* anti-thyroid peroxidase antibody, *Tg-Ab* anti-thyroglobulin antibody

## Discussion

In this study, we evaluated patients with Hashimoto’s thyroiditis, which was diagnosed based on the elevation of either anti-TPO titer or anti-Tg titer; Hashimoto’s thyroiditis patients with high titers of anti-thyroid antibodies were likely to exhibit intense diffuse FDG uptake in the thyroid. In contrast, the frequency of hypothyroidism was similar in patients with or without diffuse uptake in the thyroid.

Previous studies suggested that Hashimoto’s thyroiditis is the most frequent cause of diffuse FDG uptake in the thyroid. Yasuda et al. [10] reported that, in a sample of 36 patients with diffuse FDG uptake, there were seven with hypothyroidism and 27 who were positive for anti-TPO antibody. In a sample of 45 patients, Kim et al. [11] detected 10 patients with hypothyroidism and six with anti-TPO or anti-Tg antibodies. Karantanis et al. [12] evaluated 133 patients with diffuse uptake in the thyroid, in most of whom the indication for FDG-PET was oncology imaging, and reported that 63 (47.4%) had prior clinical diagnoses of hypothyroidism or autoimmune thyroiditis. This percentage was significantly higher than that of their control group (13/133; 9.8%). In an evaluation of 137 patients with diffuse uptake, Lee et al. [13] found that 76 (55.5%) were positive for anti-microsomal antibody. This frequency was higher than that of patients without diffuse uptake (64/1925; 3.3%).

The mechanisms underlying diffuse FDG uptake in the thyroid have not yet been clarified. Glucose utilization by normal thyrocytes appears to be dependent on TSH. However, in the current study, which evaluated only patients with Hashimoto’s thyroiditis, serum TSH and the frequency of hypothyroidism were similar regardless of diffuse FDG uptake. In fact, one patient in the present study required 100 μg levothyroxine despite low FDG uptake in the thyroid. However, Lee et al. [13] evaluated 2062 patients with or without diffuse uptake and found that TSH was significantly higher in patients with diffuse uptake. Therefore, the limited sample size in our study might underpower the results with respect to serum TSH and the frequency of hypothyroidism. Conversely, Yoshida et al. [14] evaluated 70 autopsied cases and reported that positive serum anti-thyroid antibodies in subjects without overt thyroid disease may indicate the existence of lymphocytic infiltration in the thyroid gland. In addition, inflammatory cells were shown to exhibit increased expression of glucose transporter isoforms when activated [15]. This indicates that thyroid FDG uptake may be associated with the degree of lymphocyte infiltration into the thyroid, rather than residual thyroid function, and higher SUVmax and high titers of anti-TPO antibody may indicate a higher degree of lymphocytic infiltration. Furthermore, in the current study, Hashimoto’s thyroiditis patients with high titers of anti-TPO antibody were more likely to exhibit diffusely swollen thyroids with a heterogeneous pattern on US. In a previous study, high anti-TPO titer was highly indicative of the degree of hypoechoic pattern in autoimmune thyroiditis [16]. That result is concordant with the results of the current study. Further studies are necessary to clarify the relationships between FDG uptake, the degree of chronic thyroiditis, and the degree of hypothyroidism.

Anti-TPO antibody was predictive of SUVmax in the present study. However, Karantanis et al. [12] studied 21 patients with diffuse uptake and no prior history of thyroid disease and reported no correlation between SUVmax and TSH or anti-TPO titer. The discrepancy between their results and those of the current study may be partially due to differing backgrounds of the subjects in the two studies. The present study only included patients with serologically diagnosed Hashimoto’s thyroiditis, while Karantanis et al. [12] included patients with other diseases. Several previous studies have evaluated clinical backgrounds in patients with diffuse uptake in the thyroid [10–13], but the current study is unique in that only patients with Hashimoto’s thyroiditis were included. Additionally, in the clinical setting, diffuse FDG uptake may be observed in a patient without thyroid hormone replacement therapy, when his or her anti-TPO antibody titer is high.

The primary limitation of the present study was its small sample size. Notably, the relationship between higher SUVmax and high titers of anti-TPO antibody was observed in this study. However, additional studies with larger sample sizes are required to evaluate the relationship between TSH and diffuse FDG uptake. Another limitation of the present study was the method used for clinical diagnosis of Hashimoto’s thyroiditis. We evaluated only clinical symptoms and ultrasonographic findings but did not perform fine needle aspiration, to exclude the possibility of neoplastic diseases (e.g., thyroid lymphoma).

## Conclusions

The results of the present study suggest that patients with Hashimoto’s thyroiditis and high titers of anti-thyroid antibodies are likely to exhibit intense diffuse FDG uptake in the thyroid. However, the frequency of hypothyroidism was similar in patients with or without diffuse uptake in the thyroid. Therefore, it should be borne in mind that thyroid function may be clearly impaired even in the presence of only mild FDG uptake in the thyroid.

## Abbreviations

CT: Computed tomography
FDG: ^18^F-fluorodeoxyglucose
PET: Positron emission tomography
SUVmax: Maximum standardized uptake value
Tg: Hyroglobulin
TPO: Thyroid peroxidase
TSH: Thyroid stimulating hormone
US: Ultrasonography
